# The challenges of keeping clinicians unaware of their participation in a national, cluster-randomised, implementation trial

**DOI:** 10.1186/s12910-022-00794-9

**Published:** 2022-05-30

**Authors:** Jex Kuo, Sonja Woodall, Jane Harding, Caroline Crowther, Jane Alsweiler

**Affiliations:** 1 Department of Paediatrics: Child and Youth Health, University of Auckland, Auckland Mail Centre, PO BOX 92019, Auckland, New Zealand; 2 Faculty of Science, University of Auckland, Auckland, New Zealand; 3 Liggins Institute, University of Auckland, Auckland, New Zealand

**Keywords:** Ethics, Newborn, Hypoglycaemia, Locality approval

## Abstract

**Background:**

Implementation of recommendations from clinical practice guidelines is essential for evidence based clinical practice. However, the most effective methods of implementation are unclear. We conducted a national, cluster-randomised, blinded implementation trial to determine if midwife or doctor local implementation leaders are more effective in implementing a guideline for use of oral dextrose gel to treat hypoglycaemic babies on postnatal wards. To prevent any conscious or unconscious performance bias both the doctor and midwife local implementation leaders were kept unaware of the trial. This paper reports the ethical dilemmas and practical challenges of ensuring clinicians remained unaware of their involvement in an implementation trial.

**Methods:**

We sought approval from the National Health and Disability Ethics committee to keep clinicians unaware of the trial by waiving the standard requirement for locality approval usually required for each district health board. The ethics committee did not approve a waiver of consent but advised that we approach the chief executive of each district health board to ask for provisional locality approval. Ultimately it was necessary to seek ethics approval for three separate study designs to keep clinicians unaware of the trial.

**Results:**

The median (IQR) time for chief executive approval was 16 (6–40) days and for locality approval was 57 (39–84) days. We completed 21 different locality approval forms for 27 hospitals.

**Conclusions:**

Keeping clinicians unaware of their involvement in a national implementation cluster-randomised trial is feasible. However, despite a national ethics committee, significant logistical challenges were time consuming and delayed trial completion. Co-ordination of the locality approval process would help facilitate multi-centre trials.

## Background

Evidence-based practice improves patient safety and outcomes, and reduces healthcare costs [1]. However, even when reliable evidence is available, there is often a large gap between research findings and clinical practice [2]. This can be attributed to many proximal factors, including inadequate practitioner training, a poor fit between treatment requirements and existing organisational structures, insufficient administrative support, and practitioner resistance to change [3]. Clinical Practice Guidelines provide systematically developed recommendations from well-designed research to assist health professionals to make appropriate decisions about patient care in specific clinical circumstances [4]. However, if guidelines are not used, or if health professionals are unaware of their existence, they add little value to clinical care. Therefore, evidence about effective methods of implementation of Clinical Practice Guideline recommendations is important to increase evidence-based clinical practice [5, 6].

Implementation science investigates effective strategies to implement Clinical Practice Guideline recommendations [7, 8], which often occurs at a hospital rather than an individual level [9]. In randomised trials of implementation strategies, clinician practice is being assessed, and thus awareness of the trial could modify clinicians’ behaviour and affect the outcome of the trial, known as the Hawthorne effect [10]. These trials therefore may require clinicians working at a hospital to be kept unaware not only of the intervention to which their hospital has been randomised, but of the existence of the trial itself [11]. However, clinicians not being aware of their participation in a trial raises ethical dilemmas.

Ethical approval, a key principle of medical research [12], is usually overseen by an independent committee, consisting of medical researchers, consumers, legal experts and indigenous health representatives. In many countries, in addition to an ethics committee reviewing an application, each participating institution e.g. hospital also has to give approval for the research to be undertaken at that site [13]. Clinicians are often intimately involved with research, either as the local investigator, or as a member of the local institutional review board. Thus, to maintain clinician blinding during an implementation trial, it may not be possible to seek locality approval from an institutional research review committee.

We recently conducted a national cluster-randomised trial comparing the effectiveness of midwife or medical implementation of a Clinical Practice Guideline for oral glucose gel to treat neonatal hypoglycaemia (the DesIGN trial) [14]. Here we report the challenges of keeping clinicians unaware of the existence of the implementation trial in their hospital to reduce performance bias, and of obtaining ethics and locality approvals.

## Methods

### Clinical practice guideline

The Clinical Practice Guideline for oral dextrose gel to treat neonatal hypoglycaemia was developed by a multi-disciplinary representative group in New Zealand [15]. The guideline recommended that late preterm and term babies diagnosed with neonatal hypoglycaemia be treated with 40% oral dextrose gel as first line management.

### Midwife or doctor leader to implement a national guideline in babies on postnatal wards (DesIGN): a cluster-randomised, controlled, trial

In New Zealand, babies in postnatal wards are cared for both by lead maternity carers, of whom the majority are midwives, and by paediatricians. Midwives usually provide primary neonatal care [16], whereas paediatricians become involved in care of babies with hypoglycaemia once a referral is received from the midwife for a baby with a low blood glucose concentration. Doctors would usually be approached to implement a guideline for neonatal treatment [17]. However, midwives previously have also successfully implemented guidelines [18]. When guidelines affect multiple clinical disciplines, it is unknown from which discipline the local implementation leader should be selected. We therefore conducted a national, cluster-randomised, clinical trial in New Zealand to determine if midwives or doctors are more effective local implementation leaders to implement hypoglycaemia guidelines for babies in postnatal wards [14]. All maternity hospitals which had both paediatric and midwifery staff caring for babies were eligible to participate. The primary outcome of the trial was the change in proportion of babies eligible for dextrose gel who were treated with oral dextrose gel for hypoglycaemia before and 3 months after implementation of the guideline. Maternity hospitals were randomised to having either a research doctor approach the clinical director of neonatal care at each hospital and ask them to nominate a senior medical staff member to be the local implementation leader or to having a research midwife approach the charge midwife and ask them to nominate a senior midwifery staff member to be the local implementation leader. Each local implementation leader was invited to attend an education day and given an implementation tool kit to enable them to implement the guideline at their maternity hospital.

### DesIGN trial ethics and locality approval

To prevent any conscious or unconscious performance bias both the doctor and midwife local implementation leaders were kept unaware of the trial [19]. As clinicians are the ones whose behaviour was being assessed, their knowledge of the trial could alter their approach to implementation of the guideline and affect the outcomes of the trial. It was possible that if clinicians knew that they were participating in the trial that the doctors and midwives might try and “compete” with each other to show that their discipline was the most effective. The success of keeping the leaders unaware of the trial was not measured as it would not have been possible to do this without revealing the existence of the study.

In New Zealand the National Health and Disability Ethics Committees (Ethics Committee) are responsible for reviewing all health research and checking that it meets the ethical standards of the National Ethics Advisory Committee, which aim to protect the health and rights of participating individuals [20]. Studies require Ethics Committee review if they involve human participants, the use, collection or storage of human tissue, or the use or disclosure of health information [13]. The Ethics Committees provide the option of having applications reviewed at either open or closed committee meetings. According to the operational standard for the Ethics committee, it is desirable for these meetings to be open to the public to hold accountability [13]. However, an open meeting would defeat the purpose of trying to keep doctors and midwives unaware of the randomised trial, and so a closed meeting was requested and granted.

In addition to Ethics Committee approval, locality approval from each of the participating district health boards is required before the study commences. There are 20 district health boards in New Zealand, funded from central government, each in turn is responsible for the funding and provision of health care in their regional areas. Public hospitals are owned and funded by district health boards. Because some doctors or midwives at each hospital would be involved in the trial as the local investigator, or be members of the hospital’s research committee which normally reviews applications for locality approval, we requested that the Ethics Committee waive the necessity for the locality approval of the study for the randomisation and implementation phase of the trial, and only require locality approval for the collection of the data on the proportion of eligible babies who were treated with dextrose gel use before and after the guideline implementation. While the Ethics Committee members understood the reasons for keeping clinicians unaware of the trial, they felt unable to give approval for research to happen in hospitals without locality approval, and our request for a waiver was not approved. As an alternative, we were given permission to approach the chief executive officer of each district health board and ask them individually to give consent for their district health board to participate in the randomised trial. The Chair of the Ethics Committee wrote a letter which could be distributed to the chief executive officers giving the rationale for this approach and provide assurance that this had Ethics Committee approval. Once the chief executive officer gave approval, the local implementation leader had been appointed and the guideline implemented [14], we could then seek locality approval in the usual way for data collection.

### Applications to ethics committee

To keep clinicians unaware of the randomised trial, it was necessary to make three different applications to the Ethics Committee. The first was the original Ethics Committee application for the primary protocol, for which we sought locality approval from the chief executive officer for the randomised trial to take place (15/NTA/31). However, to request locality approval for the data collection, the district health board research offices requested a copy of the original Ethics Committee application. Therefore, it was necessary to submit a second Ethics Committee application (15/NTA/135), five months after the initial Ethics Committee application, which outlined the details of the implementation and data collection, described only as an audit of oral dextrose implementation, with no details about the randomised trial. In two district health boards the chief executive officer approved the randomised trial, but the research committee declined this second protocol for guideline implementation and data collection. For the district health boards where locality approval for the second protocol was declined, we made a third Ethics Committee application (16/STH/56), twelve months after the initial Ethics Committee application, described only as an audit of oral dextrose gel use, to allow the collection of data about oral dextrose gel use for the trial intention-to-treat analysis. It took 7.5 weeks for the original Ethics Committee application to be approved, 6.5 weeks for the second application and one week for the third application. Overall, it took 13 months from the submission of the first Ethics Committee application until the approval of the third and final application.

## Results

**Fig. 1:**
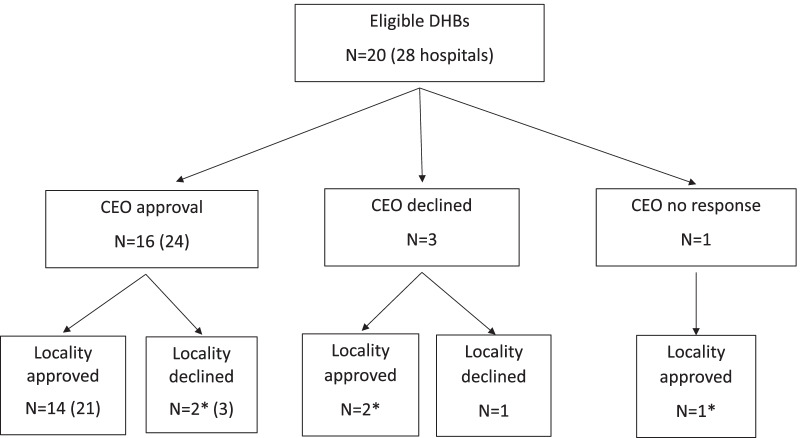
Strobe diagram for chief executive officer and locality responses. *In cases where either the chief executive officer or Clinical lead declined approval, permission to collect the data for audit only was sought. The number of hospitals it is given by the number inside the brackets if different to the number of district health boards. DHB, District health board. CEO, chief executive officer

**Fig. 2:**
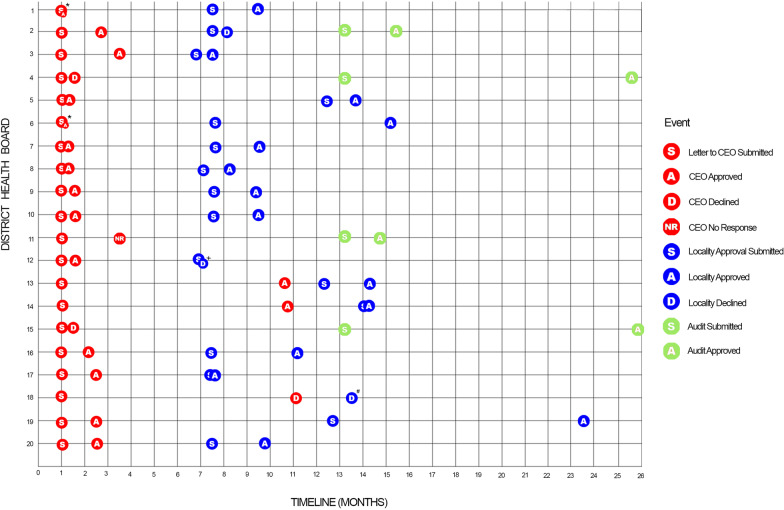
Time to ethics and locality approval for the district health boards in the DesIGN trial. *The chief executive officers from two district health boards (1 and 6) gave approval on the same day the request letter was sent. One district health board had no chief executive officer response [11]. ^+^Prior to invitation for the participation of the research, it was established the district health board was unsuitable due to technical issues, so approval for audit was not sought [12]. # Locality approval was declined, however the exact date when the locality approval was submitted was unclear [18]

We sent out letters to chief executive officers of 20 New Zealand district health boards (representing 28 maternity hospitals) asking for their approval of the randomised trial to be undertaken at their district health board, with a copy of the letter from the Ethics Committee. Of the 20 chief executive officers, 16 gave approval (24 hospitals), three declined (three hospitals) and one chief executive officer did not respond despite two follow-up requests (one hospital) (Fig. 1). Three chief executive officers asked for approval to seek advice in confidence from a medical officer not associated with the care of babies, and we agreed to this. Five chief executive officers responded within 1 week (Fig. 2). Chief executive officer approval took less than 1 month for 10/20 (50%) district health boards and longer than 6 months for 2/20 (10%) district health boards; median (IQR) 16 (6–40) days (Fig. 2). Following chief executive officer approval and randomisation we immediately approached each district health board, identified an implementation leader (either a midwife or a doctor) at each hospital and sought locality approval to implement the guideline and collect data on babies born before and after the implementation of the guideline [14]. Approval for the implementation of the guideline was given by 14/16 (87.5%) district health boards (Fig. 1). Most district health boards responded between 8 and 16 weeks after chief executive officer approval (Fig. 2). District health board approval took less than 1 month for 5/21(24%) hospitals and more than 6 months for 3/21(14%) hospitals; median 57 (39–84) days (Fig. 2). Overall, there were 21 different locality approval forms to be completed for 27 hospitals, including 5 hospitals asked only to approve collection of audit data.

## Discussion

We report the challenges encountered in conducting a national cluster-randomised, implementation trial in which we needed to keep clinicians unaware of the trial to reduce performance bias. It was possible to keep clinicians unaware of the trial’s existence by requesting chief executive officer approval for each locality, instead of the standard locality approval through each hospital’s research committee. This approach was more efficient and faster than the standard locality approval process. However, two additional ethic applications were required to maintain blinding of the participants, which added significant time to the process, taking over a year to complete. Requesting multiple locality approvals for a multicentre trial can be repetitive and consume considerable time.

Implementing a guideline successfully requires effective and evidence-based strategies [6]. Systematic reviews previously have shown that reminders, interactive educational and a multifaceted approach are the most consistently effective strategies, whereas using audit and feedback and local opinion leaders have variable effectiveness to promote behavioural change of health professionals [21, 22]. This is the first trial to describe the effectiveness of different clinical disciplines in implementing clinical practice guidelines. As the clinicians’ behaviour was being assessed, keeping the clinicians unaware of the trial was important as any knowledge of the trial may have introduced an element of competition in their behaviour. This could have biased the outcome of the trial. While previous trials of different implementation strategies have not required keeping clinicians unaware of the trial [23, 24], this approach may be necessary if further trials are planned comparing one discipline with another or other interventions where clinician awareness of the trial may alter performance. Ethics committees should consider a process to enable this to happen in a simpler and less time-consuming fashion.

A significant challenge was the ethical approval process, which was both time-consuming and complex. As the research committees at each hospital often request the original Ethics Committee application in addition to the approval letter, it was necessary to make three separate, different applications to the same ethics committee for the same study to keep clinicians unaware of the existence of the randomised trial. It took a year for the submission and approval of all 3 applications, which significantly delayed the completion of the trial. Generally, the non-standard method of gaining locality approval through chief executive officers was more efficient than the standard locality approval process. However, the response times varied widely, with the slowest chief executive officer taking 10 months to reply. In addition, the long and complicated locality approval process for multi-centre trials was challenging, with multiple different forms asking different versions of the same questions; overall, 21 different locality approval forms were filled out for 27 hospitals. Similar to our experience, an Australian study has found the majority of the time spent in obtaining ethical and site-specific approvals for multi-centre studies is on repeated and time-consuming tasks, with no added benefit for study design or participant safety [25].

The Declaration of Helsinki outlines a number of ethical principles for medical research involving human subjects, and affirms that research should be “subject to ethical standards that promote and ensure respect of all human subjects and protect their health and rights” [12]. For research to be ethical, it is generally agreed that individuals should have the freedom to make decisions for themselves about whether they want to participate or withdraw. Thus, research participants need to be fully and accurately informed of what the research entails and understand the research before any decision is made. However, the clinical staff acting as the implementation leaders in our trial were not informed about their participation in a randomised trial and their consent was not obtained or sought. Instead, they were informed that this study was an implementation audit rather than a randomised trial, which meant that clinicians were deceived about the design of the trial [26]. There appears to be a mixture of attitudes towards deception in research by both researchers and participants [27]. However, as the primary aim of our trial was to study behaviour change, participants’ knowledge of the trial may have caused bias that could have affected the study outcome; this is a valid reason why consent may not need to be sought [20]. In addition, this deception was considered not to increase risk to participants who were continuing their usual practice.

It is the duty of health researchers to ensure their research complies with appropriate standards at all times, which includes careful consideration of the ethical aspects of the research [28]. Research involving human subjects should be guided by the cardinal principles of ethics, namely autonomy, non-maleficence, beneficence and justice [29]. In our study design, the key ethical principles to be considered were autonomy and justice. Although full informed consent was not obtained, some degree of autonomy was nevertheless maintained. The implementation leaders approached by the research team at each hospital had the ability to decide to what degree, if at all, to implement the guideline, even if the chief executive officer of the district health board had already given consent. Additionally, although recommended by the study team, it was not compulsory for the implementation leaders from each hospital to be of the allocated discipline. There was a degree of freedom to choose an implementation leader from the other discipline if the clinical leader believed that person to be more effective at implementing the guideline. The risks associated with a lack of autonomy of trial participants were therefore considered to be balanced by the potential benefit of improving health outcomes by informing strategies for implementation of new guidelines in the future.

Justice in clinical research demands equitable resources for participants. As neonatal hypoglycaemia is reported more commonly at maternity hospitals in resource-poor settings [30], effective implementation of the guideline would allow hypoglycaemia to be managed consistently, which may reduce adverse outcomes. However, as the trial was conducted in a developed country, it is unknown if these findings would also be applicable in a developing country.

Cluster-randomised trials are trials in which groups of subjects are randomly allocated (as opposed to allocation of individual subjects). As a result, there are different levels at which consent can be sought which raises new ethical dilemmas [31]. Cluster randomised trials are further divided into individual-cluster and cluster–cluster type, depending on where the intervention is delivered (individual versus a unit). As our primary intervention was targeted at the level of the hospital (cluster–cluster), it was reasonable to seek consent at the level of a ‘guardian’, who was the chief executive officer in this case, without individual consent [32]. This approach to gain ethical approval is similar to a previous cluster-randomised implementation trial conducted in the United Kingdom [33].

The traditional approach to conducting multi-centre randomised trials, which requires investigators to coordinate personnel and resources across different centres as well as gaining locality approval from each individual site, can be slow and inefficient [34]. It would be beneficial to find ways to make the ethical application process more efficient, particularly for a national cluster-randomised trial requiring a multi-centre approach. In England, the Health Research Authority Approval was introduced in 2016 to incorporate all approval processes for project-based research taking place in the National Health Service (NHS), and was further extended to cover Wales in 2018. This streamlined the assessment of governance and legal compliance, with an ethical opinion by a member from the ethics committee, replaced the need for local checks of legal compliance, with only one ethics application needed, and participating NHS organisations only required to confirm their capacity and capability to undertake the study [35]. A report of the performance was published a year later, which demonstrated a significant decrease in time for the ethical approval process [36].

An alternative approach may be an amendment to the locality forms. Multi-centre studies often require multiple forms to be completed to seek locality approval, with each site using different forms, asking different versions of the same questions. This proved to be time-consuming with no clear additional benefit. It would be more efficient to have a single standard locality approval application form. If necessary, different hospitals could have site-specific questions added.

A limitation of this study is that it describes the human research ethics approval system in New Zealand, which may be different in other countries with different regulatory requirements.

## Conclusions

Despite the ethical and logistical challenges, it was possible to conduct a randomised trial in New Zealand in which the clinicians involved were unaware of its existence to reduce the risk of bias. However, ethics approval processes can be inflexible and complicated and could be facilitated by having a standardised approach to locality approval to save time and effort.

## Data Availability

Published data are available to approved researchers under the data sharing arrangements provided by the Clinical Data Research Hub, based at the Liggins Institute, University of Auckland (https://wiki.auckland.ac.nz/researchhub). Metadata, along with instructions for data access, are available at the University of Auckland’s research data repository, Figshare (https://auckland.figshare.com). Data access requests are to be submitted to Data Access Committee via researchhub@auckland.ac.nz. De-identified published data will be shared with researchers who provide a methodologically sound proposal and have appropriate ethical and institutional approval. Researchers must sign and adhere to the Data Access Agreement that includes a commitment to using the data only for the specified proposal, to refrain from any attempt to identify individual participants, to store data securely and to destroy or return the data after completion of the project. The Hub reserves the right to charge a fee to cover the costs of making data available, if required.

## Abbreviations

Ethics Committee: National Health and Disability Ethics Committees
DesIGN trial: Midwife or doctor leader to implement a national guideline in babies on postnatal wards: a cluster-randomised, controlled, trial
